# Supplementary material for: Epidemiological Study of p16 Incidence in Head and Neck Squamous Cell Carcinoma 2005–2015 in a Representative Northern European Population
Source: Cancers (Basel). 2022 Nov 21;14(22):5717. doi: 10.3390/cancers14225717 (PMC9688375; doi:10.3390/cancers14225717)
Supplement: Supplementary file 1 [file cancers-14-05717-s001.zip › cancers-2007322-supplementary.pdf]

Supplement Table S1. Five-year survival and recurrence. Recidive and residual were defined as a recurrence appearing over and under six months after end of treatment, respectively.

		N	%
Five-year survival	Living	569	55,1
	Died of HNSCC	316	30,6
	Died of other cause	148	14,3
Recurrence in five years	No	626	60,6
	Residual	192	18,6
	Recidive	215	20,8

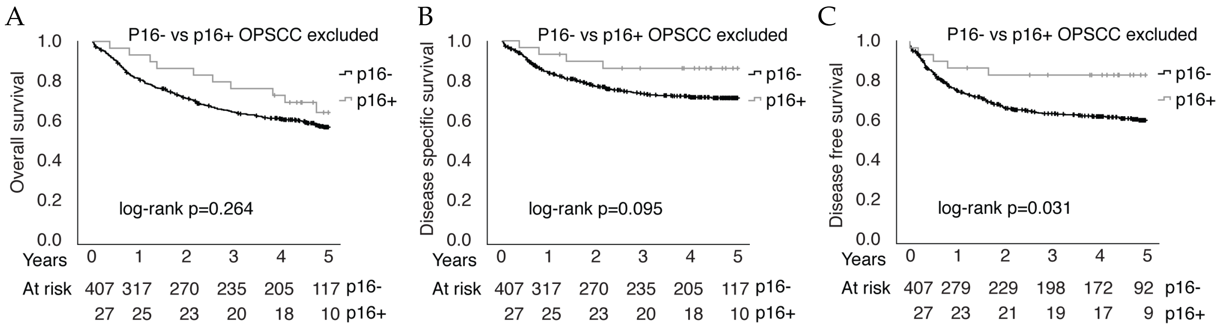

Supplement Figure S1. Overall survival (A), disease-specific survival (B) and disease-free survival (C) comparison between p16- and p16+ in HNSCC OPSCC patients excluded.

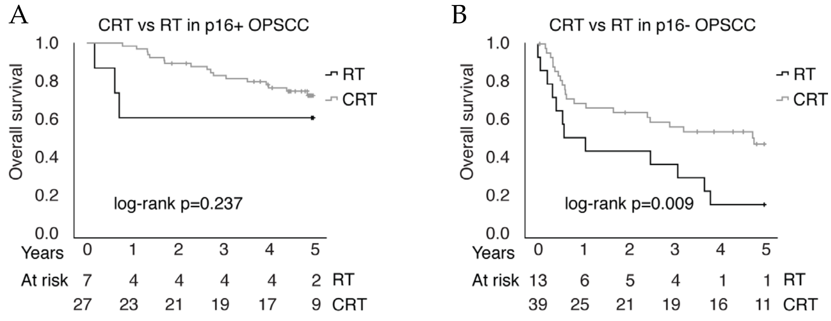

Supplement Figure S2. Overall survival comparison between RT and CRT treatments in p16+ (A) and p16- (B) OPSCC patients.
